# RWLMod—Potential Model to Study Plant Tolerance in Drought Stress Conditions

**DOI:** 10.3390/plants10122576

**Published:** 2021-11-25

**Authors:** Florin Sala, Mihai Valentin Herbei, Ciprian Rujescu

**Affiliations:** 1 Department—Soil Sciences, Compartment of Soil Science and Plant Nutrition, Banat University of Agricultural Sciences and Veterinary Medicine “King Michael I of Romania”, 300645 Timisoara, Romania; florin_sala@usab-tm.ro; 2 Department—Sustainable Development and Environmental Engineering, Compartment Remote Sensing and GIS, Banat University of Agricultural Sciences and Veterinary Medicine “King Michael I of Romania”, 300645 Timisoara, Romania; 3 Department—Management and Rural Development, Compartment Mathematics and Statistics, Banat University of Agricultural Sciences and Veterinary Medicine “King Michael I of Romania”, 300645 Timisoara, Romania; rujescu@usab-tm.ro

**Keywords:** drought stress, drying processes, mathematical model, plant hydric stress tolerance, rate of weight loss, RWLMod, water evaporation

## Abstract

Rationale: Water loss by evaporation is a normal physiological process, in order to regulate plant temperature. Under conditions of thermal and water stress, water loss is accelerated compared to normal conditions, and the response of plants is variable. In extreme cases, it can lead to wilting and death of plants. It was found that the phenomenon of water loss behaved as a pattern in different plant species, given by two functions, logistics (first part of water loss) and hyperbola (second part of water loss) in relation to a moment m, at which the rate of water loss (RWL) has reached its maximum value. Method: We studied the water loss process for a series of plant samples on different plant species (*Picea abies* L., H. Karst; *Juniperus communis* L.; *Pinus silvestris* L.; *Thuja occidentalis* L.; *Lamium purpureum* L.; *Veronica hederifolia* L.), measuring the rate of weight loss (RWL) in controlled conditions. The drying of the samples was done in identical conditions (thermo-balance, 100 °C, standard temperature for drying the plant samples) with the real-time recording of the drying time simultaneously with the water loss rate (RWL) from the plant samples. The exposure time varied, depending on each species sample, and was approximately 1000 s. Results: The experimental data was recorded at intervals of every 10 s, during the entire drying period. RWL values varied from 0.024 to 0.054 g/min at the beginning of the drying process and reached maximum values after 70–100 s, having values between 0.258 g/min and 0.498 g/min. During the drying period, this indicator presented different graphic evolutions, difficult to be described with a single function. The first segment was described by a logistic function, and the second was described by a hyperbola, resulting in a model (RWLMod) which described the real phenomenon. This model and theoretical calculation were used to quantify the water loss in a time interval and, compared with empirical dates, no significant differences were observed, which indicated an increased degree of accuracy regarding the use of this model. Recommendation and novelty of work: The novelty of the work is given by the obtained model (RWLMod), which makes possible the description of RWL over the entire time interval, and ensures a good fit with the real data. It recommends the method and model in studies of plant behaviour under stress in relation to different influencing factors.

## 1. Introduction

Water has a vital role in plant life, in relation to physiological and metabolic processes, plant nutrition, thermoregulation, plant growth and development, tolerance to stressors, etc. Knowing the dynamics of water loss in plants has multiple applications, beginning with a better understanding of the crop behaviour under conditions of water stress; we also identified technological issues on improving methods for the processing of plant products. The literature contains studies on the physiological mechanisms of water loss [1,2] on increasing tolerance regarding hydric stress [3,4,5], and on the drying process of some aromatic plants with economic possibilities [6]. In addition, the dynamics of plant humidity was studied under the influence of some external factors, such as air speed [7], or physiological and biochemical factors [8,9]. In the case of crop plants, there was a special interest regarding the increase of plant resistance to water stress and thermal factors; a number of studies have analysed physiological indices [10,11,12], water efficiency in plants [13,14], photosynthetic capacity and production quality [15,16], regarding the increased demands for food production, in the context of population growth and climate change [17,18]. There is a confirmed existence for the particular well-defined dynamic of some indicators describing humidity, and existing studies are using a mathematical characterization of these processes. Thus, the variation in humidity is behind the creation of some mathematical models, using the study of isothermal curves [19,20], or described using sigmoid curves [21]. In fact, sigmoidal curves that form a part of the mathematical model used in this paper are found in certain distinct classifications of nonlinear models used in agricultural sciences [22]. Phenomena with a downward asymptotic trend have been evaluated in studies regarding the behaviour of biological and biochemical processes [22]. Moreover, the necessity of using functions based on mathematical models which describe specific situations as accurately as possible require the adjustment and modification of classical models. Regarding old exponential models or those with limited growth, there are multiple concerns for setting functions to provide such an attribute. Power-Ricker and modified logistic function [23,24] are such examples, with applications in various fields of biology.

The problem of plant water loss was addressed in this study, in terms of rate of weight loss (RWL), under controlled conditions. By measuring the rate of water loss (RWL) in different plant species, the behaviour of the process was found according to a pattern, regardless of the species studied. A logistic function describes the first part of the process (RWL), and a hyperbolic function describes the second part of the process (RWL), in relation to the RWLmax value, recorded at all samples, but at different times. The typical approach in this study led to the finding of a mathematical model that described RWL throughout the drying process. This model (RWLMod) is the solution of this study in the description of RWL in plants. The model would facilitate the study and better understanding of the behaviour of plants (including crop plants) in conditions of water and heat stress, such approaches all the more necessary in the context of climate change.

## 2. Results

For each plant species studied, the series of data recorded in real time (Table 1, Table 2, Table 3, Table 4, Table 5 and Table 6) on the rate of weight loss (RWL) expressed in grams/minute, respectively weight (g) and drying time, are presented. These values have been obtained for the corresponding time instants of the measurements, carried out at intervals of ten seconds. Similarly, for each sample, the maximum value of RWL has also been distinctly indicated, represented as RWL_max_. The total duration of the process was specific to each sample, observing at the end of the tables the moment in time when the experiment stopped automatically, when the amount of water lost had become negligible. However, the upper limit of the time was about 1000 s. These data series were the basis for the statistical determination of the coefficients of the functional models.

Rate of weight loss has values between 0.024–0.054 g/min at the initial moment in time (t = 10 s), and the highest value was represented in the nettle. Afterwards, an increased growth rate was observed for this indicator, thus as approximately 70–100 s from the debut; RWL had values between 0.258 g/min for pine and 0.498 g/min for nettle. The limitation stage was observed at the end of this time interval and for a short period (10–20 s). The following stage regarded RWL variation; a rapid decrease on the unit of time, from the maximum values mentioned above until the values corresponding to the moment of time t of approximately 150 s, after which RWL had a slow trend of decrease until the end of the exposure period.

The following tables, respectively one for each sample (e.g., statistically calculated coefficients, corresponding to the functional model, sample “n” in Table 7, Table 8, Table 9, Table 10, Table 11 and Table 12, show for each functional model the values of the coefficients, statistically calculated, then the values rsq. (R^2^ coefficient of determination), and. sig. (significance probability) for testing the accuracy from the statistical point of view. Sample “n” indicates each plant species studied. For the first branch of the function, all values are superior to 0.990, indicating an almost perfect fitting, and all sig. values are smaller than 0.001, indicating a high degree of accuracy. In addition, for the second branch, the values rsq. are high, rsq. = 0.804 being, in fact, the lowest value. Further, for each individual sample, sig. < 0.001.

Moreover, we presented both values of water losses; on the one hand, those resulting from the theoretical integral calculations, and on the other hand, the real values obtained by real-time determinations. For each sample, separately, the results were presented distinctly, for each branch of the function. The data were statistically tested on the differences between the groups determined by the theoretical and empirical method, using the Mann–Whitney test in SPSS. The values obtained are U = 14.5, sig. = 0.575 for the time segment corresponding to the first branch (logistic model), respectively, and U = 15, sig. = 0.631 for the time segment corresponding to the second branch (hyperbole), indicating the acceptance of the null hypothesis. Therefore, it can be considered that the two data sets do not differ.

**Figure 1:**
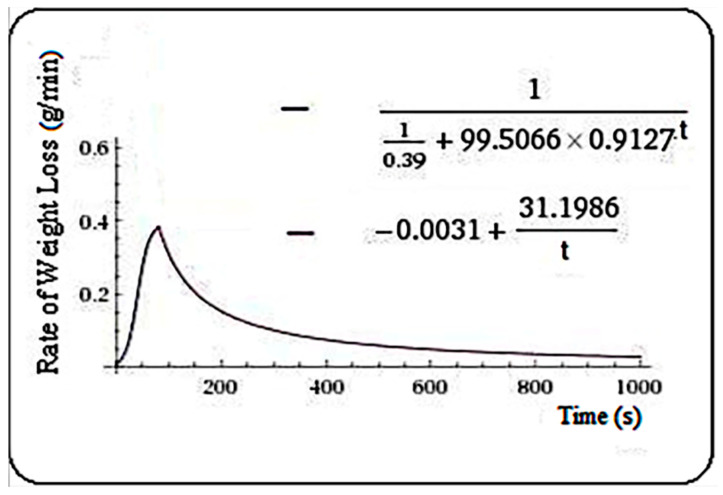
Rate of weight loss (g/min) in spruce samples.

**Figure 2:**
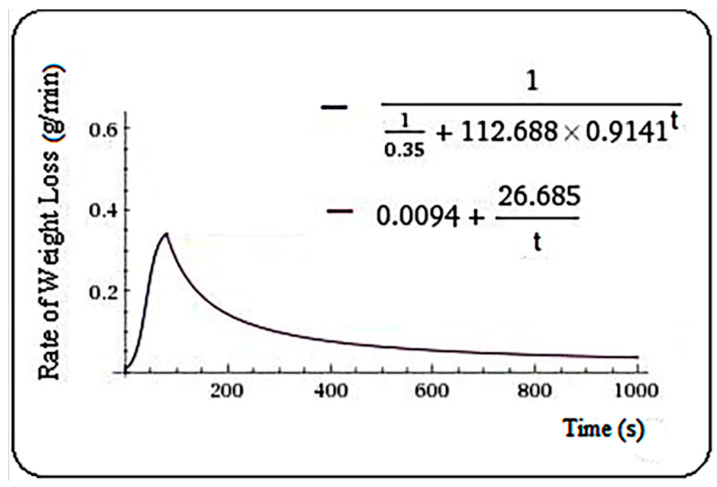
Rate of weight loss (g/min) in pine samples.

**Figure 3:**
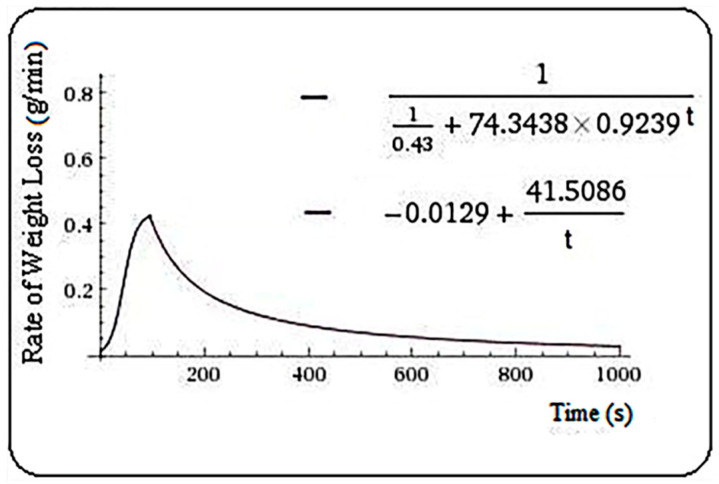
Rate of weight loss (g/min) in juniper samples.

**Figure 4:**
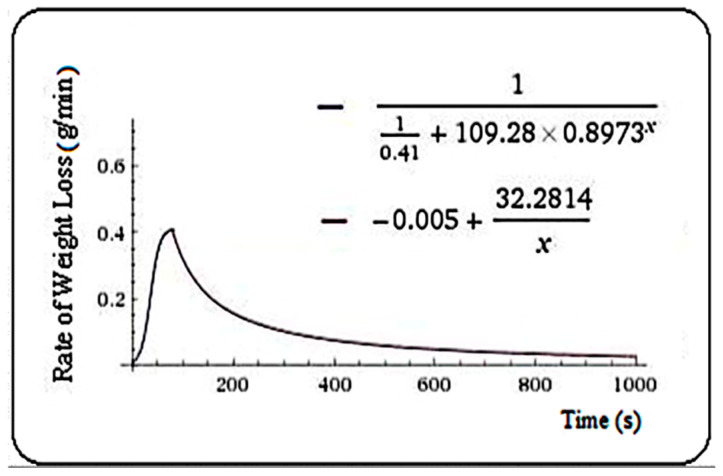
Rate of weight loss (g/min) in thuja samples.

**Figure 5:**
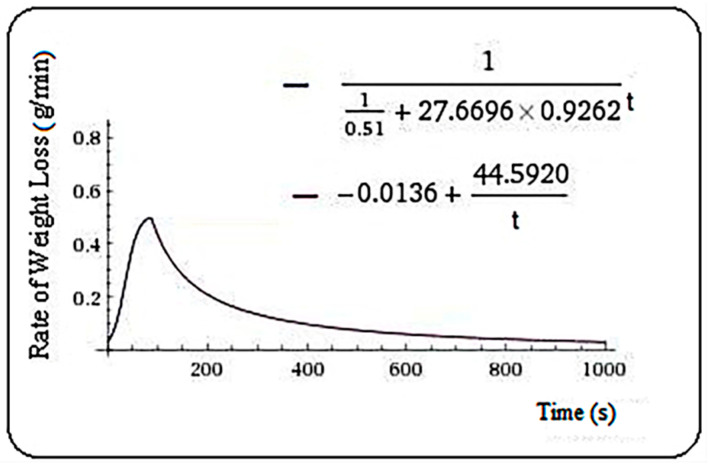
Rate of weight loss (g/min) in nettle samples.

**Figure 6:**
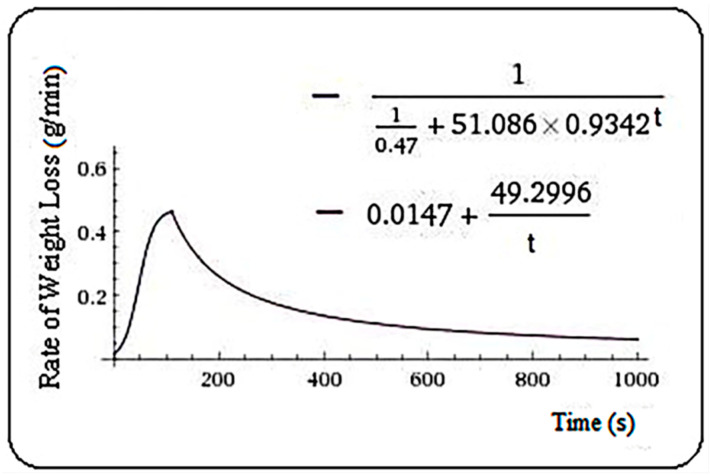
Rate of weight loss (g/min) in veronica samples.

At the end of the presentation for each sample, the expression of the functional model is represented by a graph (rate of weight loss (g/min), sample, in the Figure 1, Figure 2, Figure 3, Figure 4, Figure 5 and Figure 6. Here, the value of the theoretical maximum point (t = m) can also be observed, thus indicating the time (theoretical) when the maxim (theoretical) of RWL takes place. Thus, for the sample “spruce”, the nonlinear Equation (1) was solved, resulting in the value: t = m = 81.2 s. Thus, for the sample “spruce”, we have the Equation (2).

(1) $$\frac{1}{\frac{1}{0.39}+99.5066\cdot 0.9127^{t}}=-0.0031+\frac{31.1986}{t}$$

(2) $$f\left(t\right)=\left\{ \begin{array}{l} \frac{1}{\frac{1}{0.39}+99.5066\cdot 0.9127{\;}^{t}},\;\;\;t\leq 81.2\\ -0.0031+\frac{31.1986}{t},\;\;\;\;t>81.2 \end{array}\right.$$

Similar calculations were made solving nonlinear equations corresponding to the other samples and resulting in the functions below. For pine, Equation (3) was found, with m = 80.6 s.

(3) $$f\left(t\right)=\left\{ \begin{array}{l} \frac{1}{\frac{1}{0.35}+112.688\cdot 0.9141^{t}},\;\;\;t\leq 80.6\\ 0.0094+\frac{26.658}{t},\;\;\;\;t>80.6. \end{array}\right.$$

For the juniper sample, the resulted model is the Equation (4), and m = 92.5 s.

(4) $$f\left(t\right)=\left\{ \begin{array}{l} \frac{1}{\frac{1}{0.43}+74.3438\cdot 0.9239{\;}^{t}},\;\;\;t\leq 92.5\\ -0.0129+\frac{41.5086}{t},\;\;\;\;t>92.5 \end{array}\right.$$

For thuja, Equation (5) was found, m = 74.8 s.

(5) $$f\left(t\right)=\left\{ \begin{array}{l} \frac{1}{\frac{1}{0.41}+109.28\cdot 0.8973^{t}},\;\;\;t\leq 74.8\\ -0.005+32.2814,\;\;\;\;t>74.8 \end{array}\right.$$

Then, for nettle, the model is Equation (6), m = 86.6 s.

(6) $$f\left(t\right)=\left\{ \begin{array}{l} \frac{1}{\frac{1}{0.51}+27.6696\cdot 0.9262^{t}},\;\;\;t\leq 86.6\\ -0.0136+\frac{44.5920}{t},\;\;\;\;t>86.6 \end{array}\right.$$

Respectively, Equation (7) was found for Veronica, and m = 109.8 s.

(7) $$f\left(t\right)=\left\{ \begin{array}{l} \frac{1}{\frac{1}{0.47}+51.086\cdot 0.9342^{t}},\;\;\;t\leq 109.8\\ -0.0147+\frac{49.2996}{t},\;\;\;\;t>109.8 \end{array}\right.$$

## 3. Discussion

Multiple concerns have existed to study and describe aspects related to water in plants, or plant products, and the modeling approach has been the basis of many methods and techniques of investigation. The drying process is a method commonly used for conditioning plants. The generally accepted definition in the literature is reducing the moisture content of a certain product. The widespread use appears in preparation techniques for the medicinal herbs in beverages; in the beginning, fresh biomass (herba) will be subject to drying processes, a mandatory stage which reflects directly in the quality of the finished product, the period during which the product can be stored without quality depreciation, and also multiple economic aspects of generating profits [25].

Müller and Heindl [25] have studied the drying parameters for *Salvia officinalis.* One of the conclusions reported was related to the water activity (a_w_). It correlates with the relative humidity (RH) of the air in the areas adjacent to the material to be analysed, therefore over the limit of RH > 70%, the development of some bacteria, fungus, residue (lees) has been noticed—issues that have a direct effect on the state of product quality.

The transformations occurring at a fixed temperature are also a topic often analyzed. Thus, sorption isotherms are described mathematically for *Artemisia dracunculus* with the help of the Halsey equation [25], but also with the help of other known models: BET, Caurie, GAB, Halsey, Henderson, Lewicki, Modified Mizrahi, Oswin, Peleg [26]. In addition, extensive isotherms for a series of plants were found, such as *Salvia officiinalis* [25], *Artemisia dracunculus*, *Mentha piperita*, *Thymus vulgaris* [26,27,28], *Mentha crispa* [29]), *Mentha viridis*, *Salvia officinalis*, *Lippia citriodora* [30], *Ficus deltoidea* [31]), *Melisa officinalis* [32]), *Chenopodium ambrosioides* [33]), *Citrus sinensis* [34]), *Ziziphus spina-christi* [21]), *Phylanthus ambelica*, and *Zingiber officinale* [20]).

Furthermore, fruits are often subjected to drying processes. Simal et al. [35] and Kaya et al. [36] describe, using simulation methods, the dynamic water loss from kiwifruit; similar studies regarding various tropical fruits were conducted by Ceylan et al. [37] or Fernando and Amarasinghe [7].

This subject seems to be in full evolution at the moment, especially considering that modern food technology uses a high range of plants, for which the conducted studies are still not sufficiently detailed regarding water loss.

Rate of weight loss (RWL), defined as the amount of water lost in the time interval, can be discussed through physical analogy with the speed–vector size, thereby creating the possibility for it to be described with the help of mathematical physics equations. Jones and Sleeman [38] studied various biological models described by equations of this type.

Moreover, the rate of water loss is not constant; it is different from one species to another, from one organ of the plant to another, the way water content is present in the certain product (free water at intracellular level or present as links with various compounds).

The drying temperature, humidity and air velocity directly influence this parameter, and depending on a specific practical purpose, there are multiple studies that indicate the recommended parameters for drying in order to improve or protect some useful active principles.

Rate of weight loss of water in plants is less studied in a direct manner, but more indirectly by deduction from other calculations. However, here it has presented great interest mainly because of the different mathematical approaches that can be applied to this indicator. For example, determining the total quantity of water lost could be determined by using the properties of the definite integral.

At the same time, studying the RWL, there is the possibility of making direct observations in order to rapidly reduce the content of free water in plants. This fact can be used specifically in order to reduce the risk of developing microorganisms with undesirable effect over the quality of plant products.

In similar research to the one proposed in this study, Kaya and Aydin [39] investigated mint (*Mentha spicata* L.) and nettle (*Urtica dioica*) leaves in order to describe the loss of humidity in the presence of some variable external factors: air temperature, air velocity and relative humidity. Temperatures were lowered closer to the natural environment, with working values being 35, 45, 55 °C, and a longer drying period (30–50 min). Study regarding the leaves of lemon grass (*Cymbopogon citratus* *Stapf*) is also worth mentioning, although this was focused on the decrease of humidity, and various functional models that describe the trend shown by the experimental data were presented [40].

Theoretical mathematical models are most often made on simulated data, as a construction of the model, and will be subsequently verified in practice, on real data, in various case studies. The model proposed in this study (RWLMod) was built on real data, obtained from the six plant species studied. The model can be used in studies and in other plant species. It can also be used to describe RWL in relation to various influencing factors in plant life. The mathematical model was compared with the real distribution of the values obtained for each plant species. The fit between the mathematical model and the actual data series within each species, and the values for rsq. and sig. (as parameters of statistical safety of the fit), validates the model for the study conditions.

A direct overview on the experimental data from the present study indicated an initially ascending trend, followed by a descending part, characterized by right skewed distribution; an evolution which can be explained by physiological considerations [2]. Even if in practice mathematical modelling we find functions with approximately similar features, though in line with the trend shown by the experimental data which is the subject of this study, they present some particularities that make them different, and if these would be applied as models in this purpose, they could lead to significant errors. Even more RWL behaviour is different for the time period analysed, this being the reason why they chose to use a function consisting of two branches. The first branch, corresponding to the initial timeframe, when the plant samples show a rapid increase in the rate of water loss, is characteristic to an exponential model.

Basically, until the maximum level that occurs in a range of 80–100 s after the start, RWL increases by about 9–10 times compared to baseline. Because it is followed by a shorter range of time, a small limitation, we considered using a model based on sigmoid function, thus the phenomenon studied would be best approximated by a logistic function. For the second branch of the function, a hyperbole was used, in line with its graphic peculiarities. In the beginning, there is a rapid decrease of the water content on the time unit, in the vicinity of the maximum point, but towards the end, a very little amount of water begins to be lost even if the time interval is bigger. Specifically, in the case of each sample, towards the end of the time range studied, referring to the last 150 s of the drying process, RWL reveals low values, almost null. These aspects may be important for a better understanding of plant behaviour in extreme conditions of heat and water stress.

The chosen model (RWLMod) is a real function of a real continuous variable. In fact, the problem of continuity was placed only in one point, namely the separation point of the two time intervals. The continuity problem was solved after determining point (m) as being the intersection from the two branches of the function. In addition, this is a novelty brought by this study. The continuity immediately induces integrability of the function, and at the same time it implies the possibility to apply the formulas of integral calculation, more specifically the properties of the defined integrals regarding the determination of the area bounded by the graph of a function, to determine the amount of water lost between two given points in time. Moreover, this may be of interest for various other studies on the behaviour of plants in conditions of water and heat stress, especially in the context of climate change and the stress generated by it, for plants in general, and for crops in particular.

Performing direct comparisons on the total amount of water lost, on the one hand determined by the theoretical methods using formulas of integral calculation, and on the other hand using empirical data, there was little difference noticed, therefore insignificant. This was visible for all samples analysed, and statistical testing of the differences indicated that the values were close, and validated the proposed model (RWLMod).

## 4. Materials and Methods

Biological material was represented by different plants species, both woody trees (*Picea abies* L., H. Karst; *Juniperus communis* L.; *Pinus silvestris* L.; *Thuja occidentalis* L.), and herbaceous (*Lamium purpureum* L.; *Veronica hederifolia* L.). Fresh material, represented by leaves of species studied, was used for the determination. The tree species were about 20–25 years old. From herbaceous plants, leaf samples were taken at the flowering stage. The leaf samples were randomly harvested from the plant species studied, transported to the laboratory, and determinations were made. The humidity of the samples was variable (juniper, M = 59.71%; nettle, M = 79.16%; pine, M = 52.66%; spruce, M = 52.86%; thuja, M = 53.77%; veronica, M = 85.87%). Under the study conditions, a similar behaviour of RWL was recorded, regardless of plant species, and the samples’ humidity.

The drying process was conducted under controlled conditions with a thermal balance AXIS (model ATS 60, Gdańsk, Poland), with an accuracy of determination of ± 0.001 g. Drying temperature was 100 °C (standard drying temperature), with automatic deactivation every five consecutive determinations with minimal differences, which automatically confirmed the end of the drying process. The drying temperature used (100 °C) does not represent the real living conditions of the plants; these conditions vary from one day to another, from one location to another. This temperature was chosen precisely from the perspective of capturing, in the mathematical model, the essence of the RWL phenomenon. There were between 87 and 142 data series recorded regarding drying parameters, distinctly for each sample, at every interval of 10 s. The data was automatically recorded on a computer using the software package PROMas version 2.2.0.0, and later processed mathematically and statistically.

For this study, there were recorded parameters regarding rate of weight loss due to water evaporation (RWL) in a process of controlled drying, representing the amount of water lost in the period of time and expressed in grams/minute. It was observed directly the maximum rate of weight loss due to water evaporation (RWL_Max_), drying time (t) expressed in seconds, and weight (w) expressed in grams.

For the determination of the mathematical model (RWLMod) to describe the RWL from the plant samples considered in this study, the primary observation was that the general distribution of the RWL has several distinct graphic segments. The types of functions used to describe the phenomenon of water loss were determined starting from their particular shape which was resulted from the point clouds image. Values of the function coefficients were determined using procedure SPSS regression/curve estimation. Additionally, the values returned regarding the coefficient of determination, and also its level of statistical significance, were the basis for the confirmation of the chosen functional model. The graphic representations of the functions, determination of the intersection points between the two branches, were produced after solving some nonlinear equations, with the Wolfram Alpha application as a basis. The functions used to describe the two distinct branches of the graph of RWL are in Equation (8).

(8) $$f\left(t\right)=\{\begin{array}{c} \frac{1}{\frac{1}{u}+a\cdot b^{t}},\;\;t\in [0,m]\\ \alpha +\frac{\beta }{t},\;\;\;t\in [m,\infty ] \end{array}$$

where a, b, α, β are the coefficients of the functions and u is the upper bound.

**Figure 7:**
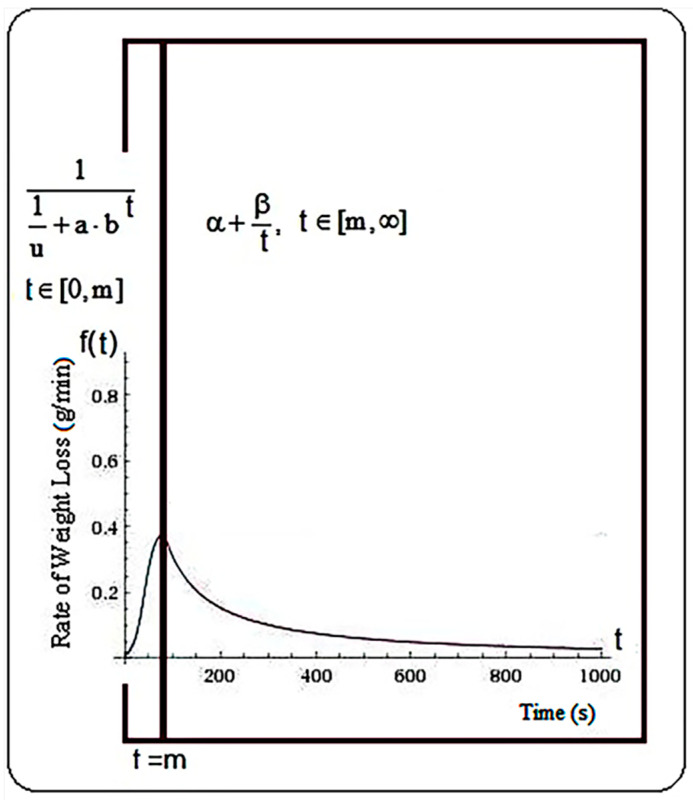
The general form of the function proposed to describe the trend of water loss.

Thus, if for x = m the maximum value of the function is obtained, first branch (logistic function) specific for the initial phase, the time interval [0, m] describes the progress made before the maximum point (m = RWL_max_). The time interval after the moment m is described by the hyperbola (Figure 7), presented in the second branch of the function (8). Continuity of the function is studied in the point t = m. Thus, to eliminate disadvantages of possible discontinuity points (points where the function is not integrable), the value of the point t = m was established as the intersection of the two functions, specifically by solving, for each sample individually, nonlinear Equation (9).

(9) $$\frac{1}{\frac{1}{u}+a\cdot b^{t}}=\alpha +\frac{\beta }{t}$$

**Figure 8:**
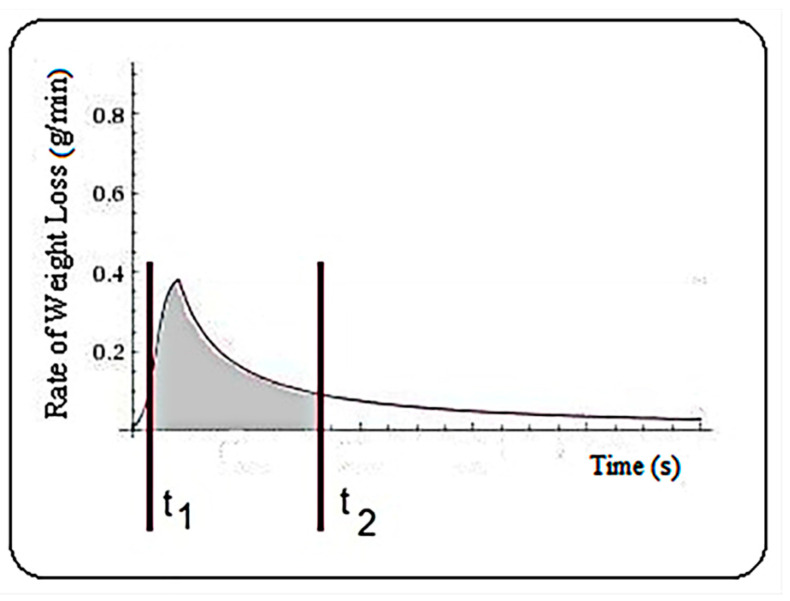
The integral calculation for the amount of water lost in the time interval t_1_, t_2_.

The total quantity of water lost in a time interval $\left[t_{1},t_{2}\right]$ represented by C (Figure 8), was determined using integral calculation, defined by the integral Equation (10).

(10) $$C=\int_{t_{1}}^{t_{2}}f\left(t\right)dt$$

Primitive functions for each branch are represented by Equation (11).

(11) $$I=\int_{}^{}f\left(t\right)dt=\int_{}^{}\frac{1}{\frac{1}{u}+a\cdot b^{t}}dt,\;J=\int_{}^{}\left(\alpha +\frac{\beta }{t}\right)dt$$

After performing the calculations, the results are described by Equations (12) and (13), respectively.

(12) $$I=-\frac{u}{\ln b}\cdot \ln\left|b^{-t}+au\right|+C_{1}$$

(13) $$J=\alpha t+\beta \ln\left|t\right|+C_{2},\;C_{1},C_{2}\in ℜ$$

**Figure 9:**
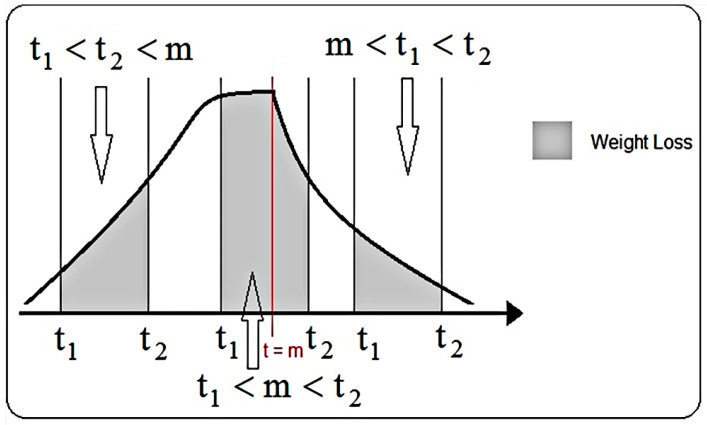
The amount of water lost in a period of time.

Regarding the positioning of the time interval compared to the maximum value, here are the cases I, II and III (Figure 9), and the quantity of water lost is described by Equations (14)–(16), respectively.

(14) $$\begin{array}{c} (I)\;t_{1}<t_{2}<m\\ C=\int_{t_{1}}^{t_{2}}f\left(t\right)dt=\int_{t_{1}}^{t_{2}}\frac{1}{\frac{1}{u}+a\cdot b^{t}}dt={\left(-\frac{u}{\ln b}\cdot \ln\left|b^{-t}+au\right|\right)|}_{t_{1}}^{t_{2}}=-\frac{u}{\ln b}\cdot \left(\ln\frac{\left|b^{-t_{2}}+au\right|}{\left|b^{-t_{1}}+au\right|}\right) \end{array}$$

(15) $$\begin{array}{c} (\mathrm{II})\;t_{1}<m<t_{2}\\ C=\int_{t_{1}}^{t_{2}}f\left(t\right)dt=\int_{t_{1}}^{m}\frac{1}{\frac{1}{u}+a\cdot b^{t}}dt+\int_{m}^{t_{2}}\alpha +\frac{\beta }{t}dt=-\frac{u}{\ln b}\cdot \left(\ln\frac{\left|b^{-m}+au\right|}{\left|b^{-t_{1}}+au\right|}\right)+\alpha \left(t_{2}-m\right)+\beta \ln\frac{t_{2}}{m} \end{array}$$

(16) $$\begin{array}{c} (\mathrm{III})\;m<t_{1}<t_{2}\\ C=t=t_{1}\int_{m}^{t_{2}}\alpha +\frac{\beta }{t}dt=\alpha \left(t_{2}-t_{1}\right)+\beta \ln\frac{t_{2}}{t_{1}} \end{array}$$

To evaluate the differences between the total amount of water lost, determined by the method of integral calculation described above and rate of weight loss determined by direct measurement, the time interval $\left[t_{1},t_{2}\right]$ used is the entire time range from the first measurement, $t_{1}=0$, to the last measurement, corresponding to each sample individually. The maximum point t = m is found within the time interval $\left[t_{1},t_{2}\right]$, so it was used in the formula (II). Determination by measuring the amount of water lost was actually determined by the type differences:$\left|{\mathrm{weight}\;(t}_{2}{)-\;\mathrm{weight}\;(t}_{1})\right|$, using the primary test data.

## 5. Conclusions

The examples taken in the study, represented by the six different plant species, herbaceous and arboreal, showed that the obtained model (RWLMod) has a high level of safety and a high degree of robustness, being able to describe RWL with a high level of probability and fit, under controlled study conditions. Although only the sensitivity of the model in the description of RWL in some plant species was considered in this study, additional studies are possible to evaluate the sensitivity and accuracy of the model to other plant species, and in relation to possible factors influencing vegetation. There is also the possibility to evaluate other parameters, in relation to which the water regime in plants is influenced, and especially the loss of water in conditions of water and thermal stress. Although it exceeds the scope of this study, the proposed model would be useful in studies in crop plants, on water loss and tolerance to thermal and water stress in relation to soil conditions, minerals, plants nutritional status, biochemical composition of plants, and with crops health status. These aspects constitute, besides studies in other plant species, for the additional experimental validation of the model, and future directions of theoretical and applied research.

## Figures and Tables

**Table 1:** Statistical data series on water loss for the sample *Picea abies* L., H. Karst (Spruce).

Time (s)	Rate of Weight Loss (g/min)	Weight (g)	Time [s]	Rate of Weight Loss (g/min)	Weight (g)	Time (s)	Rate of Weight Loss (g/min)	Weight (g)
10	0.024	2.911	160	0.198	2.321	860	0.006	1.383
20	0.054	2.902	170	0.192	2.289	870	0.006	1.382
30	0.108	2.884	180	0.192	2.257	880	0	1.382
40	0.192	2.852	190	0.192	2.225	890	0.006	1.381
50	0.282	2.805	200	0.186	2.194	900	0.006	1.380
60	0.336	2.749	210	0.186	2.163	910	0.006	1.379
70	0.366 *	2.688	220	0.186	2.132	920	0	1.379
80	0.336	2.632	230	0.180	2.102	930	0.006	1.378
90	0.300	2.582	240	0.180	2.072	940	0.006	1.377
100	0.270	2.537	250	0.180	2.042	950	0	1.377
110	0.246	2.496	260	0.174	2.013	960	0.006	1.376
120	0.228	2.458	270	0.174	1.984	970	0.006	1.375
130	0.216	2.422	280	0.168	1.956	980	0	1.375
140	0.204	2.388	290	0.168	1.928	990	0	1.375
150	0.204	2.354	300	0.168	1.9	1000	0.006	1.374
			…	…	…			

* registered value of RWL_max_.

**Table 2:** Statistical data series on water loss for the sample *Pinus silvestris* L. (Pine).

Time (s)	Rate of Weight Loss (g/min)	Weight (g)	Time (s)	Rate of Weight Loss (g/min)	Weight (g)	Time (s)	Rate of Weight Loss (g/min)	Weight (g)
10	0.024	3.017	160	0.174	2.501	1010	0.006	1.44
20	0.048	3.009	170	0.174	2.472	1020	0.006	1.439
30	0.078	2.996	180	0.168	2.444	1030	0.006	1.438
40	0.150	2.971	190	0.174	2.415	1040	0.006	1.437
50	0.252	2.929	200	0.168	2.387	1050	0	1.437
60	0.306	2.878	210	0.168	2.359	1060	0.006	1.436
70	0.324 *	2.824	220	0.162	2.332	1070	0.006	1.435
80	0.294	2.775	230	0.162	2.305	1080	0.006	1.434
90	0.258	2.732	240	0.168	2.277	1090	0.006	1.433
100	0.240	2.692	250	0.156	2.251	1100	0	1.433
110	0.216	2.656	260	0.162	2.224	1110	0.006	1.432
120	0.204	2.622	270	0.156	2.198	1120	0	1.432
130	0.192	2.59	280	0.156	2.172	1130	0.006	1.431
140	0.186	2.559	290	0.15	2.147	1140	0.006	1.43
150	0.174	2.53	300	0.15	2.122	1150	0	1.43
			…	…	…			

* registered value of RWL_max_.

**Table 3:** Statistical data series on water loss for the sample *Juniperus communis* L. (Juniper).

Time (s)	Rate of Weight Loss (g/min)	Weight (g)	Time (s)	Rate of Weight Loss (g/min)	Weight (g)	Time (s)	Rate of Weight Loss (g/min)	Weight (g)
10	0.024	3.034	160	0.21	2.344	870	0.006	1.234
20	0.054	3.025	170	0.21	2.309	880	0.006	1.233
30	0.114	3.006	180	0.198	2.276	890	0.006	1.232
40	0.204	2.972	190	0.198	2.243	900	0.006	1.231
50	0.282	2.925	200	0.192	2.211	910	0.006	1.23
60	0.336	2.869	210	0.198	2.178	920	0	1.23
70	0.378	2.806	220	0.192	2.146	930	0.006	1.229
80	0.402	2.739	230	0.186	2.115	940	0.006	1.228
90	0.420 *	2.669	240	0.192	2.083	950	0.006	1.227
100	0.39	2.604	250	0.18	2.053	960	0	1.227
110	0.336	2.548	260	0.186	2.022	970	0.006	1.226
120	0.294	2.499	270	0.18	1.992	980	0	1.226
130	0.258	2.456	280	0.18	1.962	990	0.006	1.225
140	0.240	2.416	290	0.174	1.933	1000	0	1.225
150	0.222	2.379	300	0.174	1.904	1010	0.006	1.224
			…	…	…			

* registered value of RWL_max_.

**Table 4:** Statistical data series on water loss for the sample *Thuja occidentalis* L. (Thuja).

Time (s)	Rate of Weight Loss (g/min)	Weight (g)	Time (s)	Rate of Weight Loss (g/min)	Weight (g)	Time (s)	Rate of Weight Loss (g/min)	Weight (g)
10	0.024	2.849	160	0.198	2.2	730	0.006	1.33
20	0.078	2.836	170	0.204	2.166	740	0.006	1.329
30	0.150	2.811	180	0.192	2.134	750	0.006	1.328
40	0.252	2.769	190	0.198	2.101	760	0.006	1.327
50	0.330	2.714	200	0.186	2.07	770	0.012	1.325
60	0.384	2.65	210	0.192	2.038	780	0	1.325
70	0.402 *	2.583	220	0.180	2.008	790	0.006	1.324
80	0.354	2.524	230	0.186	1.977	800	0.006	1.323
90	0.318	2.471	240	0.180	1.947	810	0.006	1.322
100	0.282	2.424	250	0.174	1.918	820	0	1.322
110	0.258	2.381	260	0.174	1.889	830	0.006	1.321
120	0.240	2.341	270	0.162	1.862	840	0.006	1.32
130	0.222	2.304	280	0.168	1.834	850	0	1.32
140	0.216	2.268	290	0.162	1.807	860	0	1.32
150	0.210	2.233	300	0.156	1.781	870	0.006	1.319
			…	…	…			

* registered value of RWL_max_.

**Table 5:** Statistical data series on water loss for the sample *Lamium purpureum* L. (Nettle).

Time (s)	Rate of Weight Loss (g/min)	Weight (g)	Time (s)	Rate of Weight Loss (g/min)	Weight (g)	Time (s)	Rate of Weight Loss (g/min)	Weight (g)
10	0.054	2.702	160	0.252	1.862	1050	0.012	0.576
20	0.138	2.679	170	0.252	1.82	1060	0.006	0.575
30	0.240	2.639	180	0.240	1.78	1070	0.006	0.574
40	0.324	2.585	190	0.240	1.74	1080	0.006	0.573
50	0.390	2.52	200	0.234	1.701	1090	0.006	0.572
60	0.438	2.447	210	0.234	1.662	1100	0.006	0.571
70	0.468	2.369	220	0.228	1.624	1110	0.006	0.57
80	0.498 *	2.286	230	0.222	1.587	1120	0.006	0.569
90	0.432	2.214	240	0.216	1.551	1130	0.006	0.568
100	0.384	2.15	250	0.216	1.515	1140	0	0.568
110	0.336	2.094	260	0.210	1.48	1150	0.006	0.567
120	0.312	2.042	270	0.204	1.446	1160	0.006	0.566
130	0.288	1.994	280	0.198	1.413	1170	0	0.566
140	0.276	1.948	290	0.198	1.38	1180	0.006	0.565
150	0.264	1.904	300	0.186	1.349	1190	0	0.565
			…	…	…			

* registered value of RWL_max_.

**Table 6:** Statistical data series on water loss for the sample *Veronica hederifolia* L. (Veronica).

Time (s)	Rate of Weight Loss (g/min)	Weight (g)	Time (s)	Rate of Weight Loss (g/min)	Weight (g)	Time (s)	Rate of Weight Loss (g/min)	Weight (g)
10	0.036	3.399	160	0.270	2.627	1280	0.006	0.49
20	0.066	3.388	170	0.264	2.583	1290	0.006	0.489
30	0.120	3.368	180	0.252	2.541	1300	0.006	0.488
40	0.198	3.335	190	0.252	2.499	1310	0.006	0.487
50	0.270	3.29	200	0.24	2.459	1320	0.006	0.486
60	0.324	3.236	210	0.246	2.418	1330	0	0.486
70	0.372	3.174	220	0.234	2.379	1340	0.006	0.485
80	0.414	3.105	230	0.240	2.339	1350	0.006	0.484
90	0.444	3.031	240	0.240	2.299	1360	0.006	0.483
100	0.462 *	2.954	250	0.228	2.261	1370	0	0.483
110	0.408	2.886	260	0.234	2.222	1380	0	0.483
120	0.366	2.825	270	0.228	2.184	1390	0.006	0.482
130	0.330	2.77	280	0.222	2.147	1400	0.006	0.481
140	0.306	2.719	290	0.228	2.109	1410	0	0.481
150	0.282	2.672	300	0.222	2.072	1420	0	0.481
			…	…	…			

* registered value of RWL_max_.

**Table 7:** Statistical coefficients calculated, corresponding to the functional model, sample “spruce”.

a	b	u	Spruce	t_2_	t_1_
99.5066	0.9127	0.39		80	10
I(t_2_)	I(t_1_)	total branch 1 (integral calculation)	total branch 1 (measurement)	Sig.	Rsq.
31.30963	15.88585	0.25	0.27	0.000	1
α	β			t_2_	t_1_
−0.0031	31.1986			1000	80
J(t_2_)	J(t_1_)	total branch 2 (integral calculation)	total branch 2 (measurement)	Sig.	Rsq.
212.4123	136.4651	1.26	1.25	0.000	0.832

Note. Sig.—significance probability; Rsq.—R^2^ coefficient of determination; a, b, α, β—coefficients of the function (8); u—upper bound; t_1_ and t_2_—time interval; I—values returned by equation (12) for the mentioned time moment; J—values returned by Equation (13) for the mentioned time moment.

**Table 8:** Statistical coefficients calculated, corresponding to the functional model, sample “pine”.

a	b	u	Pine	t_2_	t_1_
112.688	0.9141	0.35		80	10
I(t_2_)	I(t_1_)	total branch 1 (integral calculation)	total branch 1 (measurement)	Sig.	Rsq.
28.11475	14.5556	0.22	0.24	0.000	0.99
α	β			t_2_	t_1_
0.0094	26.6			1150	80
J(t_2_)	J(t_1_)	total branch 2 (integral calculation)	total branch 2 (measurement)	Sig.	Rsq.
198.6827	117.5681	1.35	1.34	0.000	0.804

**Table 9:** Statistical coefficients calculated, corresponding to the functional model, sample “juniper”.

a	b	u	Juniper	t_2_	t_1_
74.3438	0.9239	0.43		90	10
I(t_2_)	I(t_1_)	total branch 1 (integral calculation)	total branch 1 (measurement)	Sig.	Rsq.
38.83818	19.18521	0.32	0.36	0.000	0.996
α	β			t_2_	t_1_
−0.0129	41.5086			1010	90
J(t_2_)	J(t_1_)	total branch 2 (integral calculation)	total branch 2 (measurement)	Sig.	Rsq.
274.1153	185.6198	1.47	1.44	0.000	0.888

**Table 10:** Statistical coefficients calculated, corresponding to the functional model, sample “thuja”.

a	b	u	Thuja	t_2_	t_1_
109.28	0.8973	0.41		80	10
I(t_2_)	I(t_1_)	total branch1 (integral calculation)	total branch 1 (measurement)	Sig.	Rsq.
32.82901	14.62778	0.3	0.32	0.000	0.997
α	β			t_2_	t_1_
−0.005	32.2814			870	80
J(t_2_)	J(t_1_)	total branch 2 (integral calculation)	total branch 2 (measurement)	Sig.	Rsq.
214.1464	141.058	1.21	1.2	0.000	0.874

**Table 11:** Statistical coefficients calculated, corresponding to the functional model, “nettle”.

a	b	u	Nettle	t_2_	t_1_
27.6696	0.9262	0.51		80	10
I(t_2_)	I(t_1_)	total branch 1 (integral calculation)	total branch 1 (measurement)	Sig.	Rsq.
41.00061	18.55301	0.37	0.41	0.000	0.987
α	β			t_2_	t_1_
−0.0136	44.592			1190	80
J(t_2_)	J(t_1_)	total branch 2 (integral calculation)	total branch 2 (measurement)	Sig.	Rsq.
299.6035	194.3153	1.75	1.72	0.000	0.92

**Table 12:** Statistical coefficients calculated, corresponding to the functional model, sample “veronica”.

a	b	u	Veronica	t_2_	t_1_
51.086	0.9342	0.47		110	10
I(t_2_)	I(t_1_)	total branch 1 (integral calculation)	total branch 1 (measurement)	Sig.	Rsq.
51.79227	22.49395	0.48	0.51	0.000	0.990
α	β			t_2_	t_1_
0.0147	49.2996			1420	110
J(t_2_)	J(t_1_)	total branch 2 (integral calculation)	total branch 2 (measurement)	Sig.	Rsq.
378.7108	233.3488	2.42	2.4	0.000	0.823

## Data Availability

The data presented in this study are available in article.
